# Editorial: Developmental Biology and Regulation of Osteoclasts

**DOI:** 10.3389/fcell.2021.769320

**Published:** 2021-10-22

**Authors:** Yankel Gabet, Drorit Neumann, Noam Levaot, Ari Elson, Natalie A. Sims

**Affiliations:** ^1^Department of Anatomy and Anthropology, Sackler Faculty of Medicine, Tel Aviv University, Tel Aviv, Israel; ^2^Department of Cell and Developmental Biology, Sackler Faculty of Medicine, Tel Aviv University, Tel Aviv, Israel; ^3^Department of Physiology and Cell Biology, Faculty of Health Sciences, Ben-Gurion University of the Negev, Beersheba, Israel; ^4^Department of Molecular Genetics, The Weizmann Institute of Science, Rehovot, Israel; ^5^St. Vincent's Institute of Medical Research, Melbourne, VIC, Australia; ^6^Department of Medicine at St. Vincent's Hospital, The University of Melbourne, Melbourne, VIC, Australia

**Keywords:** osteoclast, osteoclastogenesis, coupling, osteopetrosis, bone disease, imaging, bone resorption

The proper structure and function of the skeleton is critical for the health and well-being of vertebrates. Bone structures enable growth, posture, movement, protect the internal organs, regulate calcium levels, and provide the proper environment for production and maturation of several types of hematopoietic cells. Bone is reshaped and renewed throughout life by the processes of bone modeling and remodeling, respectively (Sims and Vrahnas, 2014). These processes depend on the coordinated actions of mesenchymal-derived osteoblasts, which form new bone matrix, and hematopoietic-derived osteoclasts, which remove it (Sims and Vrahnas, 2014). Osteocytes, which are osteoblast lineage cells that reside within the bone matrix, and regulate the opposing activities of osteoblasts and osteoclasts in response to physiological and pathological cues (Schaffler et al., 2014).

Haemopoietic-derived osteoclasts are large, multi-nucleated phagocytic cells that are formed through fusion of monocyte/macrophage precursors. These cells are unique in the sense that they are the only cells that can resorb bone matrix. The essential nature of their activity is best demonstrated by the consequences of their absence or inactivity, which occur in various diseases. In such cases significant changes in the quality and mass of bone may ensue, severely reducing the quality of life of patients and in some cases, leading to their demise. During the process of bone remodeling, osteoclasts also produce “coupling factors,” which provide the necessary message to osteoblasts to form new bone matrix in order to replace the degraded bone and thereby maintain the structural integrity of the skeleton (Sims and Martin, 2020).

This series of articles, which we outline here, explores the development and function of osteoclasts, and examines the molecular, genetic, and environmental cues directed at these cells in order to deepen our understanding of their involvement in health and disease.

Osteoclast differentiation involves multiple steps prior to bone resorption, including induction of osteoclast-specific gene expression patterns, cell fusion, and bone surface attachment; these are highlighted in the review of Nedeva et al., which focuses on the interaction of ITAM Adaptor FcRγ with the osteoclast-associated receptor (OSCAR) in bone health and disease. This special issue also includes a review of the role of bone morphogenetic proteins (BMPs) in the interaction between osteoblasts and osteoclasts (Lademann et al.). While the roles of BMPs in promoting bone formation are well-established (Sampath and Reddi, 2020), this review stresses that BMPs also have direct and indirect actions on the osteoclast lineage to stimulate osteoclast formation, bone resorption and osteoclast-derived coupling signals that promote bone formation. The review also considers the impact of this novel aspect of BMP2 function when using recombinant BMP in the clinic for bone repair and regeneration. In parallel, Rashed et al. review the literature demonstrating that the cytokine RANKL, the key regulator of osteoclast differentiation, also promotes bone formation through “outside-in” signaling within the osteoblast. Their work indicates that this pathway can be activated via RANKL-binding proteins and suggests a novel approach to stimulating bone formation. An additional review by Inoue et al. summarizes the field of miRNA regulation of both bone resorption and coupling factor activity.

Osteoclastogenesis is critical for maintaining bone architecture and integrity. While excessive bone resorption by osteoclasts is associated with osteoporosis and rheumatoid arthritis, impaired osteoclast differentiation and activity lead to osteopetrosis, a severe and often fatal condition (Sobacchi et al., 2013). Mutations in Sorting Nexin 10 (SNX10) were recently identified in patients with malignant infantile osteopetrosis. Elson et al. provide an in-depth discussion of SNX10 and its roles in intracellular membrane trafficking and link these roles to vital processes controlling osteoclast functions. They also point to a link between membrane trafficking in osteoclasts with the majority of genes associated with recessive osteopetrosis. Together with a review by Ribet et al. on the function of a range of membrane transport proteins, these articles stress the critical importance of membrane trafficking and membrane transport proteins to osteoclast function, and provide an update on the wide range of membrane transport proteins now known to regulate osteoclast activity. Further emphasizing the importance of cytoskeletal mobility in osteoclasts, Delaisse et al. review studies that demonstrate the dynamics of osteoclasts that “glide” over the resorbed bone by continuous reorganization of their ruffled border and associated enzymes, while maintaining a tightly sealed resorption compartment.

Pertaining to the controversial skeletal role(s) of erythropoietin (EPO) in bone (Hiram-Bab et al., 2017), Suresh et al. now add a novel aspect to resolve this conflict at least partially. Their work distinguishes between physiological levels of EPO, which maintain bone homeostasis by regulating the balance between bone formation and adipogenesis in bone marrow stromal cells, and pharmacological administration of exogenous EPO at higher levels, which decreases bone marrow adipogenesis, stimulates osteoclast activity and induces bone loss. Hypoxia inducible factors (HIFs), which also control EPO production, are important determinants of bone homeostasis via direct and indirect effects on osteoclasts (Meng et al.) in the hypoxic bone environment. HIF inhibitors may thus present clinical relevance as they may target osteoclast activation and secondary bone loss in numerous diseases. Future studies on HIF signaling and its role in osteoclastogenesis will likely lead to effective treatments for human diseases involving disruption of bone homeostasis. In line with the importance of oxidative stress in osteoclast development, Oh et al. present original data on Sestrin2, an antioxidant that regulates reactive oxygen species, autophagy, and inflammation. Interestingly, these authors show that Sestrin2 has an oxidative stress-independent role in bone homeostasis by facilitating protein-protein interaction between TRAF6 and p62 and induction of NFATc1 expression during osteoclast differentiation.

While the link between osteoblast differentiation and bone marrow adipogenesis is well established (Pierce et al., 2019), the connection between marrow adipogenesis and osteoclast formation has been more controversial. In this issue, Madel et al. show that loss of adipocytes results in increased osteoclast activity that links adipocytes to osteoclasts in mice and humans. Using three different models, the authors elegantly establish a mechanism in which adiponectin signaling inhibits osteoclastogenesis *in vivo*.

Du et al. suggest that the loss of trabecular bone in a mouse tail-suspension model may be caused by reduced leukemia inhibitory factor (LIF) expression by osteocytes and loss of its known actions to stimulate bone formation. Tail suspension also results in increased osteoclast numbers, and Du et al. show that LIF directly stimulates osteoclastogenesis *in vitro*. A direct effect of LIF on osteoclast differentiation is surprising, as it contradicts earlier work that reported the absence of LIF receptor expression on osteoclasts (Allan et al., 1990).

Two original studies present novel imaging and analytical tools relevant for osteoclast research. Pánczél et al. present two elegant fluorescence-based real-time imaging and analytical assays to monitor osteoclast development *in vitro* by replacing membrane-targeted tdTomato with eGFP, under control of the osteoclast-specific cathepsin K promoter linked to the Cre recombinase gene (Ctsk-Cre). A somewhat similar approach has been used by others for intravital imaging of osteoclasts (McDonald et al., 2021). Cohen-Karlik et al. use artificial intelligence and the power of machine learning to establish an unbiased, automatic method for evaluating osteoclast number and surface in cultures.

Collectively, this series of manuscripts provides a wealth of knowledge about this unique and intriguing cell type, including new developments in understanding their formation and function. We hope it will provide a useful resource both for researchers well-versed in osteoclasts and those who are new to the field, and will stimulate new interdisciplinary research in this field.

## Author Contributions

NL and YG wrote the first draft of the manuscript. All authors contributed to manuscript revision, read, and approved the submitted version.

## Conflict of Interest

The authors declare that the research was conducted in the absence of any commercial or financial relationships that could be construed as a potential conflict of interest.

## Publisher's Note

All claims expressed in this article are solely those of the authors and do not necessarily represent those of their affiliated organizations, or those of the publisher, the editors and the reviewers. Any product that may be evaluated in this article, or claim that may be made by its manufacturer, is not guaranteed or endorsed by the publisher.
